# The prevalence and risk factors of chronic low back pain among adults in KwaZulu-Natal, South Africa: an observational cross-sectional hospital-based study

**DOI:** 10.1186/s12891-021-04790-9

**Published:** 2021-11-15

**Authors:** Morris Kahere, Themba Ginindza

**Affiliations:** Discipline of Public Health Medicine, School of Nursing and Public Health, University of KwaZulu-Natal, 2nd Floor George Campbell Building, Mazisi Kunene Road, Durban, 4041 South Africa

**Keywords:** Chronic low back pain, Prevalence, Risk factors

## Abstract

**Background:**

Globally, chronic low back pain (CLBP) is the leading cause of disability associated with economic costs. However, it has received little attention in low-and-middle-income countries. This study estimated the prevalence and risk factors of CLBP among adults presenting at selected hospitals in KwaZulu-Natal.

**Methodology:**

This cross-sectional study was conducted among adults aged ≥18 years who attended the selected hospitals in KwaZulu-Natal during the study period. A self-administered questionnaire was used to collect data on socio-demographic, work-related factors, and information about CLBP. The SPSS version 24.0 (IBM SPSS Inc) was used for data analysis. Descriptive statistics were used for demographic characteristics of participants. CLBP risk factors were assessed using multivariate logistic regression analysis. A *p*-value of ≤0.05 was deemed statistically significant.

**Results:**

A total of 678 adults participated in this study. The overall prevalence of CLBP was 18.1% (95% CI: 15.3 – 21.3) with females having a higher prevalence than males, 19.8% (95% CI: 16.0 – 24.1) and 15.85% (95% CI: 11.8 – 20.6), respectively. Using multivariate regression analysis, the following risk factors were identified: overweight (aOR: 3.7, 95% CI: 1.1 – 12.3, *p* = 0.032), no formal education (aOR: 6.1, 95% CI: 2.1 – 18.1, *p* = 0.001), lack of regular physical exercises (aOR: 2.2, 95% CI: 1.0 – 4.8, *p* = 0.044), smoking 1 to 10 (aOR: 4.5, 95% CI: 2.0 – 10.2, *p* < 0.001) and more than 11 cigarettes per day (aOR: 25.3, 95% CI: 10.4 – 61.2, *p* < 0.001), occasional and frequent consumption of alcohol, aOR: 2.5, 95% CI: 1.1 – 5.9, *p* < 0.001 and aOR: 11.3, 95% CI: 4.9 – 25.8, *p* < 0.001, respectively, a sedentary lifestyle (aOR: 31.8, 95% CI: 11.2 – 90.2, *p* < 0.001), manual work (aOR: 26.2, 95% CI: 10.1 – 68.4, *p* < 0.001) and a stooped sitting posture (aOR: 6.0, 95% CI: 2.0 – 17.6, *p* = 0.001).

**Conclusion:**

This study concluded that the prevalence of CLBP in KwaZulu-Natal is higher than in other regions, and that it is predicted by a lack of formal education, overweight, lack of regular physical exercises, smoking, alcohol consumption, sedentary lifestyle, manual work, and a stooped posture.

**Supplementary Information:**

The online version contains supplementary material available at 10.1186/s12891-021-04790-9.

## Background

Globally, chronic low back pain (CLBP) is the leading cause of disability, affecting all age groups from children to the elderly and is associated with high economic costs [1]. The healthcare costs and disability associated with chronic low back pain are projected to increase in the next decade, particularly in low-and-middle-income countries (LMIC) including Africa, where the health systems are insubstantial to deal with the increasing burden. Low back pain (LBP) is nearly a universal human experience, accounting for one-third of all daily outpatients visits, which is second from the common cold, an upper respiratory tract infection [2]. Low back pain is also ranked the third most common reason for surgical procedures and the fifth ranking cause of hospital admissions [3]. The development of CLBP from acute episodes of LBP occurs only in a limited proportion of individuals, approximately 10 to 20% [4]. Despite the relatively low prevalence of CLBP, the economic impact of this sub-category is substantial. A population-based study by Linton et al. [5] reported that 6% of CLBP cases was responsible for 41% of the total healthcare visits. Another study by the Quebec Task Force for Spinal Disorders reported that 7% of individuals with chronic low back pain accounted for 76% of all compensation costs [6].

A systematic survey of the global adult population showed that the point prevalence, 1-month prevalence, annual prevalence, and the lifetime prevalence of non-specific chronic low back pain (NCLBP) were 12, 23, 38 and 40% respectively [7–10]. Anderson et al. [3] reported a rise in the prevalence of CLBP from 3.9% in 1992 to 10.2% in 2006. Similarly, Freburger et al., reported the CLBP prevalence of 10.2% among adults in California [11]. According to Klauber et al. [12], the annual prevalence of NCLBP was 26% in 2010 among the Germany adults, which was comparatively higher than what was reported by Anderson et al. in 2006 and Freburger et al. in 2009. Similarly, Noormohammadpour et al. [13], reported a prevalence of CLBP of 27.18% among Iranian adults aged 30 to 70 years. Hartvigsen et al. [14] reported that in 2015, the global point prevalence of chronic disabling back pain was 7.3%. The CLBP prevalence of 15.4% was observed in the general Japanese adult population [15] and the 13.4% observed in Wales [16]. According to Hartvigsen et al. [14], from 1990 to 2015 the years lived with disability caused by low back pain increased by 54%, with the highest burden seen in LMIC.

Most LBP studies in Africa are work-related or occupation-based describing the incidence, prevalence, risk factors or disabilities associated with LBP on work status. Chronic low back pain is poorly investigated and often described as a sub-heading in studies investigating other musculoskeletal disorders and not as the main outcome of interest. A 2018 systematic review by Morris et al. [17] investigating the prevalence of low back pain in Africa showed a pooled lifetime, 12-months and point prevalence of low back pain of 47, 57 and 39% respectively. Another systematic review by Louw et al. [18] reported a point, annual and lifetime prevalence among adults of 32, 50 and 62% respectively. Another study by Sikiru et al. [19] investigated the prevalence of low back pain among nurses in Africa found that, the overall annual prevalence of LBP was of 70%, with females showing a higher prevalence than the males, 67.5 and 32.5%, respectively. Chronic low back pain has been poorly investigated in LMICs, including Africa. This has been necessitated by the more pressing issues of communicable diseases, which includes HIV/AIDS and the current COVID-19 pandemic [14], in this context. COVID-19 impacted hugely on the healthcare system with economic recession, multiple healthcare consequences and ultimately an anticipated rise in the prevalence of CLBP due to intervention focus shift and funding directions directed towards the COVID-19 pandemic [20].

Non-specific chronic low back pain, defined as pain or discomfort in the area below the lower 12th rib and the inferior gluteal fold, lasting for at least 12 weeks, with no identifiable specific spinal disease, radiculopathy or nerve root pain [2]. Its clinical manifestations includes sacroiliac joint pain, facet/zygapophyseal joint pain, and lumbosacral myofascitis [2]. Patients with low LBP can be put into two categories according to (i) the duration of symptoms (as acute with symptoms lasting less than 6 weeks, sub-acute with symptoms lasting more than 6 weeks but less than 12 weeks or chronic with symptoms lasting for at least 12 weeks) [21] and (ii) aetiological mechanism (as specific with known specific cause such as fracture, tumours, infections, inflammation, arthritis, radiculopathy or as non-specific with no identifiable cause) [22, 23]. About 80-90% of CLBP is non-specific, complicated and difficult to treat [24]. The main goal in managing low back pain is to reduce pain, improve/restore function and avoid reoccurrence, with more recommendations inclining towards minimally invasive conservative management and rehabilitation. In most cases, pharmacologic therapy is the first line of treatment while interventional therapy is considered after conservative management and medication have failed.

Almost 80% of LBP cases resolve within a few weeks with only about 20% developing chronic disabling symptoms. The disability and high economic costs caused by LBP are associated with this small percentage of CLBP sufferers, yet it is poorly investigated. Chronic low back pain has been given several different definitions in literature with some authors using different definitions over time, which makes the available evidence difficult to generalise its distribution. This results in deficiency of accurate knowledge due to the incomparability nature of studies caused by a lack of a standard definition of CLBP in terms of its anatomical characterisation and duration of symptoms. Therefore, an accurate measure of the distribution of CLBP, with estimates on prevalence, incidence, its associated risk factors, and economic burden is urgently needed to ensure a sufficient allocation of healthcare resources to address its growing public health problem. This is part of a large study that seeks to determine the burden of CLBP among adults in KwaZulu-Natal. The purpose of this study was to determine the prevalence and risk factors of CLBP among adults in, KwaZulu-Natal, South Africa.

## Methods

### Aim

The main aim of this study was to determine the prevalence and risk factors of chronic low back pain among adults in KwaZulu-Natal, South Africa.

### Study design, setting and participants

We conducted an observational cross-sectional health-facility-based study utilising a standardised self-administered questionnaire. The study was conducted in five randomly selected provincial public hospitals in KwaZulu-Natal, South Africa. A simple random sampling using the hat method was used where the first five out of all 18 hospital names to be picked were selected for inclusion. Adults’ participants aged 18 years and above who attended for healthcare services at the selected study sites during the study period and were willing to sign the informed consent were recruited into the study. Systematic random sampling technique was used to recruit participants. Those that did not satisfy the criteria for inclusion were excluded, including those who had no capacity to give consent, children, those that were mentally ill, physically disabled, those with congenital anomalies such as cerebral palsy, ambulatory issues, and other serious medical ailments. A detailed description of the sampling procedure and the sample size estimation is found in the published protocol [25].

### Sample size estimation

To estimate the prevalence of chronic low back pain, assuming the desired level of confidence (95%), an acceptable margin of error of 5% and an estimated prevalence of chronic low back pain i.e. 50% (maximum variability given unknown prevalence), a sample size of 384 was required. The sample was increased by a margin of 10% to account for potential contingencies such as non-response and it was further multiplied by the design effect (D) of 1.5. The final sample size of the study was the estimated at 650 participants.

### Study area

This study was conducted in eThekwini District Municipality in KwaZulu-Natal province, a South African east-coastal province with a population of approximately 11,074,800 people, which is about 19.6% of the total South African population and covers the geographical area of 94,361 km2. The province consists of about 19% of the youth aged 15–24 years, 28% of the middle-aged adults (25–44 years), 13% aged 45–64 years and only about 5% consists of the elderly aged over 65 years, with a 49:51 male-to-female ratio [26, 27]. KwaZulu-Natal has three provincial borders (Eastern Cape, Free State and Mpumalanga provinces) and three international borders with Mozambique, Eswatini and Lesotho.

### Data collection

Data were collected using structured standardised questionnaires which gathered baseline data on detailed information concerning the participants demographics (age, gender), socioeconomic status (highest educational level, annual household income, employment status), lifestyle factors (exercise frequency, smoking attitude, alcohol consumption), postural habits (type of work, i.e. sedentary, semi-sedentary or manual work, sitting posture), clinical (BMI, which was calculated using self-reported height and weight, chronic disease), low back pain (previous history, current status, duration, onset, severity, progression, healthcare service utilisation and social implications). Data were entered and stored into IBM SPSS statistical software version 24.0 for Windows. Each participant was assigned a unique study identification number that was used to link the questionnaire with the electronic database/sheet. Personal information blinded for researchers was kept on-site for results feedback and validation of data.

### Data analysis overview

The data were analysed using the IBM SPSS statistical software version 24.0 for Windows (IBM Corp., Armonk, NY, USA). Demographic data were analysed using descriptive statistics which were presented as frequencies (n) and proportions (%) along with their 95% confidence intervals (CI) for all variables (Tables 1 and 2). Odds ratios (ORs) and 95% confidence intervals (CIs) for CLBP biomechanical risk factors were estimated using multivariate logistic regression analysis, with adjustments made for factors significantly associated with CLPB at a significant cut-off of *p* ≤ 0.2 in the univariate analysis [27], using a dichotomous “presence or absence of CLBP” as the dependent variable. The association of pregnancy and CLBP was calculated in a separate regression model that included only the female gender in the analysis. An adjusted *p*-value of < 0.05 was deemed statistically significant.

**Table 1:** Socio-demographic characteristics of study participants (*N* = 678)

Variable	Frequency (***N*** = 678)		Chronic low back pain (***n*** = 123)	
	(n.)	(%)	(no.)	(%)
**Age**				
18 – 27	45	6.6	6	4.9
28 – 37	150	22.1	21	17.1
38 – 47	202	29.8	35	28.5
48 – 57	186	27.4	34	28.5
58+	95	14.0	27	22.0
**Gender**				
Male	284	41.9	45	36.6
Female	394	58.1	78	63.4
**Marital status**				
Single	113	16.7	20	16.3
Married	325	47.9	65	52.9
Separated	184	27.1	28	22.8
Widowed	56	8.3	10	8.1
**Body mass index (BMI)**				
Underweight	87	12.8	7	5.7
Normal	219	32.3	12	9.8
Overweight	216	31.9	62	50.4
Obese	156	23.0	42	34.1
**Highest level of education**				
No formal education	137	20.0	52	42.3
Primary	215	31.7	33	26.8
Secondary	179	26.4	24	19.5
Tertiary	147	21.7	14	11.4
**Exercise frequency**				
Yes	321	47.3	30	24.4
No	357	52.7	93	75.6
**Smoking attitude**				
No	425	62.7	30	24.4
Light smokers	140	20.6	32	26.0
Heavy smokers	113	16.7	61	49.6
**Alcohol consumption**				
No	328	48.4	30	24.4
Occasional	191	28.2	30	24.4
Frequent	159	23.5	63	51.2
**Type of work**				
Semi sedentary	396	58.4	10	8.1
Sedentary	107	15.8	43	35.0
Manual labour	175	25.8	70	56.9
**Sitting posture**				
Straight back	159	23.5	19	15.4
Stooped	114	16.8	40	32.5
Backward inclination	192	28.3	51	41.5
Forward inclination	213	31.4	13	10.6
**Use of back support**				
Yes	171	25.2	17	13.8
No	507	74.8	106	86.2
**How long in this occupation?**				
< 2	52	7.7	9	7.3
2 – 3	87	12.8	10	8.1
3 – 4	116	17.1	14	11.4
4 – 5	128	18.9	18	14.6
5+	295	43.5	72	58.5
**Positive family history of CLBP**				
Yes	326	48.1	79	64.2
No	352	51.9	44	35.8

**Table 2:** Prevalence of chronic low back pain (*N* = 678)

Age (years)	Prevalence of CLBP Males		Prevalence of CLBP Females		Overall Prevalence of CLBP	
	*no.*	*% (95%CI)*	*no.*	*% (95%CI)*	*no.*	*% (95%CI)*
18 - 27	4	13.8 (3.9 – 26.3)	2	12.5 (1.6 – 38.4)	6	13.3 (5.1 – 26.8)
28 – 37	4	6.4 (1.8 – 15.5)	17	19.5 (11.8 – 29.4)	21	14.0 (8.9 – 20.6)
38 – 47	16	19.3 (11.4 – 29.4)	19	16.0 (9.9 – 23.8)	35	17.3 (12.4 – 23.3)
48 - 57	12	15.6 (8.3 – 25.6)	22	20.2 (13.1 – 29.0)	34	18.3 (13.0 – 24.6)
58+	9	28.1 (13.8 – 46.8)	18	28.6 (17.9 – 41.4)	27	28.4 (19.6 – 38.6)
***Overall***	***45***	***15.9 (11.8 – 20.6)***	***78***	***19.8 (16.0 – 24.1)***	***123***	***18.1 (15.3 – 21.3)***

## Results

### Demographic characteristics of the participants

Out of 700 questionnaires distributed to participants between October 2019 and December 2019, only 678 were successfully completed yielding a response rate of 96.9%. Table 1 presents an outline of the sociodemographic characteristics of the participants. The ratio of males to females was approximately 2:3 (41.9%: 58.1%) (*p* < 0.001). There were 14.0% of the elderly and only 6.6% of the young adults. Most of the participants were middle-aged adults of the working class. Almost half (47.9%) of the participants were married. Approximately a third of of the participants were overweight (31.9%) and 23% were obese. Twenty percent of the participants had no formal education. There was almost a 1:1 (47.3%: 52.7%) ratio among those who performed regular exercises and those who do not exercise. A little more than half (58.4%) of the participants were semi-sedentary workers, whose work involved a mixture of office work, walking, standing, bending, and twisting. Only 23.5% of the participants reported a straight back sitting posture. Only a quarter (25.2%) of the participants used a back support when sitting. Nearly half (48.1%) of the participants had a prior evidence of CLBP in the immediate family.

### Prevalence of chronic low back pain

**Fig. 1:**
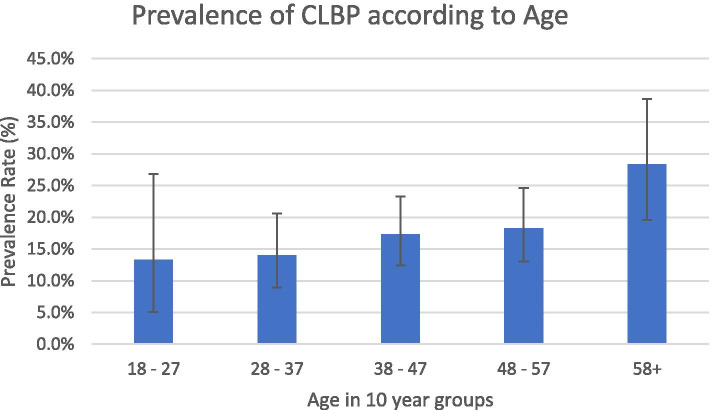
Prevalence of CLBP according to age only

As shown in Table 2, the overall prevalence of chronic low back pain was 18.1% (95% CI: 15.3 – 21.3). Females had an overall higher prevalence of chronic low back pain than males, 19.8% (95% CI: 16.0 – 24.1) and 15.9% (95% CI: 11.8 – 20.6) respectively. However, stratified by gender and age, males showed a slightly higher prevalence (13.8%) among the young adults aged 18 – 27 years compared to their females counterparts (12.5%). Similarly, the prevalence of chronic low back pain among male adults in the age category 38 – 47 was higher as compared to their female counterparts, 19.3 and 16.0%, respectively. The overal prevalence of chronic low back pain increased with increasing age (Fig. 1) with the elderly showing the highest prevalence compared to the young adults, 28.4% (95% CI: 19.6 – 38.6) and 13.3% (95% CI: 5.1 – 26.8), respectively. As shown in Tables 3 and 4, the prevalence of chronic low back pain was highest among the heavy smokers (54.0.0%), followed by the sedentary workers (40.2%), manual labourers (40.0%), frequent alcohol abusers (39.6%,), those with no formal education (38.0%,) and those with a stooped posture (35.1%).

**Table 3:** Risk factors for chronic low back pain (*N* = 678)

Characteristics	No. (%)	Univariate		Multivariate	
		COR (95%CI)	***p***-value	AOR (95%CI)	***p***-value
Age					
18 – 27	45 (6.6%)	1 (ref)		1 (ref)	
28 - 37	150 (22.1%)	1.1 (0.4 – 2.8)	0.910	3.3 (0.5 – 19.9)	0.197
38 – 47	202 (29.8%)	1.4 (0.5 – 3.5)	0.516	3.9 (0.7 – 22.5)	0.127
48 -57	186 (27.4%)	1.5 (0.6 – 3.7)	0.433	4.6 (0.8 – 26.2)	0.087
58+	95 (14.0%)	2.6 (1.0 – 6.8)	0.055	5.0 (0.8 – 31.2)	0.088
Gender					
Male	284 (41.9%)	1 (ref)		1 (ref)	
Female	394 (58.1%)	0.3 (0.9 – 2.0)	0.188	1.1 (0.5 – 2.2)	0.806
Marital Status					
Single	113 (16.7%)	1 (ref)			
Married	325 (47.7%)	1.2 (0.7 – 2.0)	0.594		
Separated	184 (27.1%)	0.8 (0.5 – 1.6)	0.573		
Widowed	56 (8.2%)	1.0 (0.4 – 2.3)	0.980		
Body Mass Index (BMI)					
Under weight	87 (12.8%)	1 (ref)		1 (ref)	
Normal	219 (32.3%)	0.7 (0.3 – 1.7)	0.404	0.4 (0.1 – 1.7)	0.229
Overweight	216 (31.9%)	4.60 (2.01 – 10.52)	0.000	3.7 (1.1 – 12.3)	0.032*
Obese	156 (23.0%)	4.2 (1.8 – 9.9)	0.001	2.3 (0.7 – 8.0)	0.186
Highest level of education					
No formal education	137 (20.2%)	5.8 (3.0 – 11.1)	0.000	6.1 (2.1 – 18.1)	0.001*
Primary education	215 (31.7%)	1.7 (0.9 – 3.4)	0.108	2.6 (0.9 – 7.8)	0.077
Secondary education	179 (26.4%)	1.5 (0.7 – 3.0)	0.279	1.4 (0.5 – 4.0)	0.529
Tertiary education	147 (21.7%)	1 (ref)		1 (ref)	
Exercise frequency					
No	357 (52.7%)	3.4 (2.2 – 5.3)	0.000	2.2 (1.0 – 4.8)	0.044*
Yes	321 (47.3%)	1 (ref)		1 (ref)	
Smoking attitude					
No	425 (62.7%)	1 (ref)		1 (ref)	
Yes – 1 to 10 cigarettes	140 (20.6%)	3.90 (2.27 – 6.71)	0.000	4.52 (2.00 – 10.24)	0.000**
Yes – 11 and above	113 (16.7%)	15.45 (9.15 – 26.09)	0.000	25.32 (10.40 – 61.23)	0.000**
Alcohol consumption					
No	231 (34.1%)	1 (ref)		1 (ref)	
Yes – occasional	252 (37.2%)	1.85 (1.07 – 3.18)	0.026	2.49 (1.06 – 5.88)	0.037*
Yes – frequently	195 (28.8%)	6.52 (3.99 – 10.66)	0.000	11.28 (4.93 – 25.83)	0.000**
Type of work					
Semi sedentary	396 (58.4%)	1 (ref)		1 (ref)	
Sedentary	107 (15.8%)	25.93 (12.41 – 54.21)	0.000	31.83 (11.23 – 90.23)	0.000**
Manual labour	175 (25.8%)	25.73 (12.82 – 51.66)	0.000	26.22 (10.05 – 68.41)	0.000**
Sitting posture					
Straight back	138 (17.5%)	1 (ref)		1 (ref)	
Stooped	126 (16.0%)	3.98 (2.15 – 7.36)	0.000	5.96 (2.02 – 17.57)	0.001*
Forward inclination	198 (25.2%)	2.67 (1.50 – 4.74)	0.001	2.54 (0.94 – 6.88)	0.067
Backward inclination	216 (27.4%)	0.48 (0.23 – 1.00)	0.051	0.44 (0.14 – 1.36)	0.152
Use of back support					
Yes	171 (21.7%)	1 (ref)		1 (ref)	
No	507 64.4%)	2.40 (1.39 – 4.13)	0.020	1.26 (0.48 – 3.29)	0.641
How long in this job					
< 2	52 (7.7%)	1 (ref)			
2 – 3	87 (12.8%)	0.62 (0.23 – 1.65)	0.337		
3 – 4	116 (17.1%)	0.66 (0.26 – 1.63)	0.364		
4 – 5	128 (18.9%)	0.78 (0.33 – 1.87)	0.581		
5+	295 (43.5%)	1.54 (0.72 – 3.32)	0.267		
Positive family history of CLBP					
Yes	326 (48.1%)	2.24 (1.49 – 3.36)	0.000	1.61 (0.82 – 3.17)	0.168
No	352 (51.9%)	1 (ref)			

Bold *p*-values on multivariate analysis were statistically significant

*Sig at *p* < 0.05

**Sig at *p* < 0.001

**Table 4:** Risk factors of chronic low back pain among females (*n* = 394)

		Females			
Characteristics		Univariate		Multivariate	
	No. (%)	COR (95%CI)	***p***-value	AOR (95%CI)	***p***-value
No. of pregnancies	394 (58.1%)	1.8 (1.4 – 2.3)	0.000	2.4 (1.4 – 4.1)	**0.000**
Education					
Primary education	123 (31.2%)	1.6 (0.7 – 3.9)	0.293	0.1 (0.02 – 0.5)	**0.003**
Secondary education	106 (26.9%)	1.3 (0.5 – 3.3)	0.570	0.1 (0.03 – 0.4)	**0.001**
Exercise Frequency					
No	207 (52.5%)	3.3 (1.9 – 5.7)	0.000	3.3 (1.1 – 10.1)	**0.036**
Smoking attitude					
Yes – 11 and above	61 (17.5%)	19.7 (10.0 – 38.6)	0.000	81.9 (17.7 – 378.5)	**0.000**
Alcohol					
Yes – frequently	98 (24.9%)	5.6 (3.0 – 10.5)	0.000	21.5 (5.6 – 83.0)	**0.000**
Type of work					
Sedentary	58 (14.7%)	22.5 (9.0 – 56.2)	0.000	51.8 (10.2 – 263.8)	**0.000**
Manual labour	106 (26.9%)	25.4 (10.9 – 59.0)	0.000	40.4 (9.4 – 174.6)	**0.000**
Sitting posture					
Stooped	69 (17.5%)	5.7 (2.3 – 14.3)	0.000	35.7 (4.6 – 268.3)	**0.001**
Forward inclination	127 (32.2%)	4.3 (1.8 – 10.3)	0.001	14.0 (2.2 – 91.6)	**0.006**

### Risk factors for chronic low back pain

In order to identify the biomechanical risk factors associated with chronic low back pain, we carried out a multivariable binary logistic regression analysis. All variables that were significant at the threshold of *p* ≤ 0.2 in the univariate analysis were selected for inclusion into the final multivariate model. The results of the multivariable regression analysis are shown in Table 3. Based on the multivariable analysis, the following risk factors were identified: overweight (aOR: 3.7, 95% CI: 1.1 – 12.3), no formal education (aOR: 6.1, 95% CI: 2.1 – 18.1), lack of regular physical exercises (aOR: 2.2, 95% CI: 1.0 – 4.8), smoking more than 11 cigarettes per day (aOR: 25.3, 95% CI: 10.4 – 61.2), frequent consumption of alcohol (aOR: 11.3, 95% CI: 4.9 – 25.8), a sedentary lifestyle (aOR: 31.8, 95% CI: 11.2 – 90.2), manual work (aOR: 26.2, 95% CI: 10.1 – 68.4) and a stooped sitting posture (aOR: 6.0, 95% CI: 2.0 – 17.6), which were positively associated with CLBP. The adjusted odds ratio of smoking more than 11 cigarettes per day (aOR: 25.3) were more than six times the odds of smoking less than 10 cigarettes per day (aOR: 4.5). Similarly, frequent alcohol consumption (aOR: 11.3) was nearly five times more likely to predict CLBP than occasional alcohol consumption (aOR: 2.5). Interestingly, the odds of sedentary lifestyle (aOR: 31.8) were higher than the odds of manual labour (aOR: 26.2) in predicting CLBP.

A separate logistic regression analysis for females only was conducted, to determine the association of pregnancy with CLBP. After multivariable adjustments, results presented in Table 4 show that, pregnancy was significantly associated with CLBP (aOR: 2.4, 95% CI: 1.4 – 4.1).

## Discussion

The aim of this cross-sectional study was to determine the prevalence and risk factors associated with CLBP among adults in KwaZulu-Natal province at selected public hospitals. The results of this study showed that the overall prevalence of chronic low back pain among adults was 18.1%. Stratified by gender, this study shows that the prevalence of CLBP among males and females were 15.9 and 19.3%, respectively. The multivariable regression analysis showed that the main predictors of CLBP among adults were overweight, lack of formal education, lack of regular physical exercises, cigarette smoking, frequent alcohol consumption, sedentary lifestyle, manual labour, and a stooped sitting posture.

The prevalence of CLBP observed in our study is comparatively higher than the 15.4% observed in the general Japanese adult population [15], the 13.4% observed in Wales [16] and the 10.2% among adults in California [11]. On the other hand, Noormohammadpour et al. [13], reported a prevalence of CLBP of 27.18% among Iranian adults aged 30 to 70 years which was comparatively higher than the present study. However, the comparability of these studies is difficulty due to the differences in methodologies. More homogenous studies are needed to be able to make a good comparison and draw conclusions and recommendations.

Stratified by gender, the present study showed a higher prevalence of CLBP among females 19.8% than males 15.9%. This is consistent with the findings of several other studies which showed an overall higher prevalence of CLBP among females than males [28, 29]. Interestingly, the present study showed that males of the age category 38 – 47 years, had a higher CLBP prevalence than their female counterpart, 19.3 and 15.9%, respectively. This could have been influenced by a multiple of factors which include, a higher proportion of male smokers, alcoholics, and manual workers in that age category than females.

According to this study pregnancy was a significant predictor of CLBP. This is consistent with the findings of several studies which reported similar findings [30–36].

Based on the multivariate analysis, this study showed that increasing age was not a significant predictor of CLBP. This is contrary to the findings of other cross-sectional studies [3, 15, 16, 28, 29].. On the other hand to this finding, Knauer et al. [29], reported that the prevalence of CLBP decreased in the elderly population. .

In the present study, overweight was identified as significant predictor of CLBP. This is consistent with the findings of Iizula et al. [15]. These findings can be explained by the postural theorem which postulate that, increasing the virtual weight of the lumbar spine, increases the load and stress/strain of the surrounding anatomical structures leading to microtrauma that will build up over time culminating in chronic disabling pain.

This study identified no formal education as a significant predictor of CLBP. Dionne et al. [37], Hagen et al. [38] and Jonsdottiir et al. [16] reported similar findings. This can be attributed to low health awareness associated with low formal education attainment. Additionally, individuals with no formal education have a higher likelihood to be engaged in manual occupations characterised by notable frequency of bending, twisting, and lifting of heavy objects, all of which have been reported to be highly associated with CLBP.

This study showed that lack of regular exercise was a significant predictor of CLBP. Similar results have been observed in previous studies [39–41]. Lack of regular exercise results in weakened myofascial structures of the back and incorrect biomechanics of the body.

Increased smoking was shown to be a significant predictor of CLBP. This is consistent with the results of similar studies [13, 42–46]. Several possible explanations have been used to clarify this association. First, smoking reduces bone mineral content, increasing the risk of osteoporosis and micro-injuries of the vertebral body resulting in an accelerated degenerative process of the spine [44, 45]. Second, smoking increases coughing, which increases the intradiscal and intra-abdominal pressures resulting in an increased risk of disc herniation [47, 48]. Lastly, smoking reduces blood flow to the disc which ultimately affect the metabolic balance of the disc resulting in disc degeneration [47, 49]. However, studies on this topic using prospective methods are required.

The current study showed that excessive and frequent consumption of alcohol was a significant predictor of CLBP. This is in line with the results found by Palacios-Ceña et al. [28].

Our study showed that a sedentary lifestyle and manual labour were both significant predictors of CLBP. These findings were consistent to a similar cross-sectional study by Jia et al. [50] and Cho et al. [51] reported that prolonged sitting without a back support is a significant predictor of LBP [51, 52]. This might be due to the fact that sitting increase the virtual weight of the lumbar spine resulting in increased strain on the surrounding anatomical structures which may results in microinjuries, scar tissue formation and fibrosis [53].

### Implications of this study

The results of this study are useful for future research focus. The prevalence of CLBP is high, and an action plan needs to be taken now to avoid the projected consequences of the increasing burden of CLBP. This study found that lack of formal education is associated with the occurrence of CLBP. This means, there is need for the creation of social support groups, health education and promotion by community health workers to those that are unable to read and understand health literature. Lack of regular exercise was also a significant predictor of CLBP. This is a wake-up call to community leaders to implement social sporting events to encourage regular physical exercises. The geriatric population should also be incorporated into the community sporting events to encourage a healthy lifestyle for all age groups. Cigarettes smoking and excessive consumption of alcohol were also significant predictors of CLBP. There is need for regular awareness campaigns, implementation of policies that discourage smoking and alcohol consumption among the young adults. Sedentary lifestyle was also a significant predictor of CLBP. All employers should be obliged to make sure that their employees have comfortable chairs, with an ergonomic lumbar support. The health and safety policy should also take into consideration implications of sitting on an uncomfortable chair for prolonged period. All workplaces should, therefore, be inspected regularly by a qualified and registered health and safety officer to make sure these safety measures are always observed in the same manner people are taking precautionary measures against COVID19.

### Limitations

The main limitation of this study was that it was done in only one region in South Africa, therefore the results of this study cannot be generalised. The other limitation was that the multivariable analyses only controlled for known confounders, without unknown confounders such as genetic factors, work demands, family demands or chronic stress. Additionally, this study was conducted in a hospital setting targeting individuals attending the hospital for healthcare services. This implies that, these participants had some ailments which could have inflated the prevalence and risk factors of CLBP. A large-scale population-based study is needed that targets households to give accurate prevalence estimates and risk factors. The other limitation of this study was the use of self-reported data; therefore it is difficult to rule out recall-bias which could have led to over or underestimation of the prevalence. A further limitation relates to the cross-sectional study design which makes it impossible to draw any inferences of causality.

## Conclusion

In summary, this study found that the prevalence of CLBP is high and that CLBP is predicted by lack of formal education, being overweight, smoking cigarettes, excessive alcohol consumption, sedentary lifestyle, manual labour, and a stooped sitting posture.

## Supplementary Information

**Additional file 1.**

## Data Availability

All data generated or analysed during this study will be included in the published article.
