# Supplementary material for: The prevalence and risk factors of chronic low back pain among adults in KwaZulu-Natal, South Africa: an observational cross-sectional hospital-based study
Source: BMC Musculoskelet Disord. 2021 Nov 15;22:955. doi: 10.1186/s12891-021-04790-9 (PMC8591969; doi:10.1186/s12891-021-04790-9)
Supplement: Supplementary file 1 — Additional file 1. [file 12891_2021_4790_MOESM1_ESM.docx]

Supplementary file 1

| Characteristics | Females | | | | |
| --- | --- | --- | --- | --- | --- |
|  | **No. (%)** | **COR (95%CI)** | **p-value** | **AOR (95%CI)** | **p-value** |
| Age |  |  |  |  |  |
| 18 – 27 | 16 (4.1%) | 1 (ref) |  |  |  |
| 28 - 37 | 87 (22.1%) | 1.70 (0.35 – 8.20) | 0.509 |  |  |
| 38 – 47 | 119 (30.2%0 | 1.33 (0.28 – 6.33) | 0.720 |  |  |
| 48 -57 | 109 (27.7%) | 1.77 (0.37 – 8.37) | 0.471 |  |  |
| 58+ | 63 (16.0%) | 2.80 (0.58 – 13.58) | 0.201 |  |  |
| Marital Status |  |  |  |  |  |
| Single | 55 (14.0%) | 1 (ref) |  |  |  |
| Married | 183 (46.4%) | 0.98 (0.46 – 2.08) | 0.957 |  |  |
| Separated | 120 (30.5%0 | 0.95 (0.43 – 2.12) | 0.897 |  |  |
| Widowed | 36 (9.1%) | 1.14 (0.41 – 3.19) | 0.799 |  |  |
| Body Mass Index (BMI) |  |  |  |  |  |
| Under weight | 54 (13.7%) | 1 (ref) |  | 1 (ref) |  |
| Normal | 137 (34.8%) | 0.69 (0.22 – 2.16) | 0.523 | 0.58 (0.08 – 4.31) | 0.597 |
| Overweight | 118 (29.9%) | 4.66 (1.72 – 12.63) | 0.003 | 3.79 (0.59 – 24.18) | 0.159 |
| Obese | 85 (21.6%) | 4.32 (1.54 – 12.09) | 0.005 | 4.45 (0.62 – 31.85) | 0.137 |
| No. of pregnancies |  |  |  |  |  |
|  |  | 1.81 (1.40 – 2.34) | 0.000 | 2.39 (1.40 – 4.09) | **0.000** |
| Education |  |  |  |  |  |
| No formal education | 82 (20.8%) | 8.50 (3.64 – 19.86) | 0.000 | 0.37 (0.09 – 1.51) | 0.164 |
| Primary education | 123 (31.2%) | 1.61 (0.66 – 3.89) | 0.293 | 0.10 (0.02 – 0.45) | **0.003** |
| Secondary education | 106 (26.9%) | 1.31 (0.52 – 3.33) | 0.570 | 0.10 (0.03 – 0.41) | **0.001** |
| Tertiary education | 83 (21.1%) | 1 (ref) |  | 1 (ref) |  |
| Exercise Frequency |  |  |  |  |  |
| No | 207 (52.5%) | 3.25 (1.87 – 5.66) | 0.000 | 3.30 (1.08 – 10.07) | **0.036** |
| Yes | 187 (47.5%) | 1 (ref) |  | 1 (ref) |  |
| Smoking attitude |  |  |  |  |  |
| No | 244 (61.9%) | 1 (ref) |  | 1 (ref) |  |
| Yes – 1 to 10 cigarettes | 81 (20.6%) | 2.34 (1.12 – 4.88) | 0.023 | 2.15 (0.62 – 7.43) | 0.225 |
| Yes – 11 and above | 61 (17.5%) | 19.71 (10.01 – 38.56) | 0.000 | 81.86 (17.71 – 378.45) | **0.000** |
| Alcohol |  |  |  |  |  |
| No | 181 (45.9%) | 1 (ref) |  | 1 (ref) |  |
| Yes – occasional | 115 (29.2%) | 1.80 (0.91 – 3.53) | 0.090 | 3.77 (0.99 – 14.40) | 0.052 |
| Yes – frequently | 98 (24.9%) | 5.64 (3.02 – 10.52) | 0.000 | 21.54 (5.59 – 83.04) | **0.000** |
| Type of work |  |  |  |  |  |
| Semi sedentary | 230 (58.4%) | 1 (ref) |  | 1 (ref) |  |
| Sedentary | 58 (14.7%) | 22.49 (9.00 – 56.20) | 0.000 | 51.79 (10.17 – 263.80) | **0.000** |
| Manual labour | 106 (26.9%) | 25.38 (10.91 – 59.04) | 0.000 | 40.41 (9.36 – 174.55) | **0.000** |
| Sitting posture |  |  |  |  |  |
| Straight back | 73 (18.5%) | 1 (ref) |  | 1 (ref) |  |
| Stooped | 69 (17.5%) | 5.70 (2.28 – 14.29) | 0.000 | 35.70 (4.75 – 268.31) | **0.001** |
| Forward inclination | 127 (32.2%) | 4.34 (1.83 – 10.29) | 0.001 | 14.04 (2.15 – 91.58) | **0.006** |
| Backward inclination | 125 (31.7%) | 0.39 (0.12 – 1.29) | 0.123 | 0.90 (0.11 – 7.52) | 0.919 |
| Use of back support |  |  |  |  |  |
| Yes | 102 (25.9%) | 1 (ref) |  | 1 (ref) |  |
| No | 292 (74.1%) | 1.76 (0.94 – 3.31) | 0.077 | 1.26 (0.34 – 4.69) | 0.733 |
| How long in this job |  |  |  |  |  |
| < 2 | 22 (5.6%) | 1 (ref) |  |  |  |
| 2 – 3 | 46 (11.7%) | 0.72 (0.20 – 2.51) | 0.602 |  |  |
| 3 – 4 | 79 (20.1%0 | 0.49 (0.15 – 1.63) | 0.247 |  |  |
| 4 – 5 | 69 (17.5%) | 0.65 (0.20 – 2.11) | 0.469 |  |  |
| 5+ | 178 (45.2%) | 1.12 (0.39 – 3.20) | 0.838 |  |  |
| Family history of CLBP |  |  |  |  |  |
| Yes | 181 (45.9%) | 1.59 (0.96 – 2.61) | 0.070 | 1.04 (0.39 – 2.79) | 0.937 |
| No | 213 (54.1%) | 1 (ref) |  | 1 (ref) |  |
